# Editorial: Biorisk management, laboratory acquired infections and clinical containment, volume II

**DOI:** 10.3389/fpubh.2024.1368828

**Published:** 2024-02-19

**Authors:** Kashif Ali, Esmeralda Meyer, Furqan Kabir

**Affiliations:** ^1^Department of Biosciences, Faculty of Life Sciences, Shaheed Zulfikar Ali Bhutto Institute of Science and Technology, Karachi, Pakistan; ^2^Department of Research Compliance and Regulatory Affairs, Institutional Animal Care and Use Committee, Emory University, Atlanta, GA, United States; ^3^Infectious Diseases Research Laboratory (IDRL), Department of Pediatrics and Child Health, Aga Khan University, Karachi, Pakistan

**Keywords:** biosafety, biosecurity, biorisk management, laboratory infections, personal protective equipment (PPE)

This particular collection is the second volume of the Research Topic entitled “*Biorisk management, laboratory acquired infections and clinical containment*.” The first volume of the series (Ali et al.) presented novel advancements in the areas of biosafety, biosecurity, gene-editing, and international instruments relevant to biosafety. Additionally, it explored the influence of virtual training and tailored tools, such as a modified hand washing regimen designed for settings with limited resources. The results presented in volume I of this series demonstrate that the utilization and accountability for correctly using biosafety and personal protective equipment (PPE) standards were successful in ensuring infection control. These measures effectively reduced the likelihood of cross-infection, particularly between healthcare providers and patients.

Following the COVID-19 pandemic, the dissemination of high consequence infections and the occurrence of contagious diseases posed a significant worldwide health security concern, underscoring the ongoing significance of biorisk management as a crucial public health issue. The preceding research underscored the challenges faced by resource-constrained nations in adhering to biosafety and biosecurity protocols, particularly during the COVID-19 pandemic. However, financial, cultural, and managerial obstacles continue to hinder compliance in many countries. Therefore, it is imperative to prioritize the promotion of biorisk management awareness and understanding to foster continuous and enhanced efficacy.

Furthermore, the preceding compilation acknowledged the obstacles that impede the effective execution of biosecurity protocols, encompassing not only the human domain but also the animal sector. Biosecurity methods are extensively recommended in the poultry industry to mitigate the prevalence of microbial infections and communicable diseases, particularly in high-risk poultry and poultry-related products. These procedures aim to minimize both the incidence of illness and death rates. Hence, it is imperative to enhance adherence to biosecurity protocols on both domestic and global scales in order to safeguard and counteract potential biological hazards.

Taking into account these factors and drawing from the previous compilation, the primary objective of volume II Research Topic is to contribute to the existing body of research in the field of biorisk management. Specifically, the aim is to evaluate about laboratory acquired infections and enhance public health. This area of concern is of significant importance as it continues to be inadequately documented and overlooked within this particular domain.

The effectiveness of antibiotic susceptibility testing (AST) is contingent upon the expertise and proficiency of laboratory personnel. Limited access to knowledge and skills is a prevailing issue in numerous low- and middle-income countries (LMICs), such as Pakistan. Hence, the primary aim of a research by Saeed et al. was to utilize open-access online courses as a means to enhance the expertise of laboratory personnel engaged in the identification and documentation of antimicrobial resistance (AMR). A total of seven online modules were created, consisting of 22 courses, with the specific objective of enhancing the laboratory detection capabilities for antimicrobial resistance (AMR). A cohort of 227 individuals from Pakistan was enrolled over the period of March 2018 to June 2020. The courses on AST and biosafety exhibited the highest number of registered participants and the most notable completion rate, whereas courses pertaining to quality-related topics garnered comparatively less attention. The results indicated a statistically significant improvement in 13 out of the 20 courses tested, with a *p*-value < 0.05. The study findings revealed a significant difference in course completion rates between participants from public and private sector laboratories. The findings of this study indicate that open online courses (OOCs) hold significant promise in addressing knowledge gaps in the field of AST related to laboratory settings with limited resources.

Respiratory viral infections (RVIs) are a significant public health issue, and prior research has yielded conflicting results about the efficacy of mask-wearing in preventing respiratory viral infections (RVIs). Consequently, a comprehensive review and meta-analysis were undertaken by Chen et al., to examine the efficacy of mask-wearing. Various databases were searched to identify relevant studies assessing the efficacy of mask-wearing. The effectiveness of mask-wearing in avoiding respiratory viral infections (RVIs) was assessed using the risk ratio (RR) in randomized controlled trials (RCTs) and cohort studies, while case-control studies utilized the odds ratio (OR). A total of 31 studies, including a participant pool of 13,329 individuals, met the criteria for inclusion in the meta-analyses. In general, the findings indicated that the utilization of masks was efficacious in mitigating respiratory viral infections (RVIs). The sensitivity analysis demonstrated that the findings of the meta-analyses were resilient and trustworthy. The meta-analysis of case-control studies and the majority of subgroup analyses did not exhibit any substantial indication of publication bias. In order to mitigate the danger of respiratory viral infections (RVI), individuals are advised to use masks when venturing into public spaces.

The working atmosphere and perception of security among healthcare staff have been significantly altered by the COVID-19 outbreak. Medical laboratory scientists encountered novel occupational challenges as well. Laboratorians were assigned the responsibility of conducting innovative examinations for SARS-CoV-2, while lacking awareness of the potential hazards involved. In addition, during the initial stages of the pandemic, individuals experienced significant levels of stress due to the implementation of stringent sanitary measures and the apprehension surrounding the contraction of the novel virus. A study by Wolszczak-Biedrzycka et al. was aimed to examine the degree to which this cohort of healthcare practitioners adjusted to novel working circumstances 1 year following the onset of the pandemic. The research was undertaken during the onset of the fourth wave of the pandemic in Poland, specifically spanning from September 10 to October 31, 2021. The study examined the challenges and anxieties encountered by this particular cohort of professionals at the onset of the pandemic, along with the alterations in their perspectives throughout subsequent surges of the COVID-19 virus. The data analysis revealed that a majority of medical laboratory scientists had adapted to the ongoing pandemic and the subsequent alterations in their work environment by the onset of the fourth wave. The study further suggests that, alongside the provision of sufficient personal protective measures, it is imperative to bring awareness to mental health and offer health care personnel emotional support during times of high stress such as a pandemic.

The presence of sufficient laboratory capacity plays a crucial role in the effective execution of a comprehensive surveillance system for antimicrobial resistance (AMR). A study by Moirongo et al. assessed the laboratory infrastructure and antimicrobial resistance (AMR) monitoring practices in health facilities in Kenya, with a focus on identifying their capacities and limitations. A sample of healthcare facilities, encompassing both public and commercial sectors, was selected using a convenience sampling method, representing various locations throughout the country. Data was collected utilizing online surveys administered to laboratory managers. The assessment encompassed many aspects related to the quality assurance, management, and dissemination of antimicrobial resistance (AMR) data, materials, and equipment. It also included considerations for staffing, microbiology expertise, biosafety measures, and certification. The primary deficiencies observed in AST facilities were inadequate availability of laboratory information management technology (LIMT) and limited adoption of external quality assessment (EQA) initiatives for diagnostic cultures. The availability of laboratory technology was much higher in urban regions compared to rural areas, indicating higher than 2-fold difference. Laboratories that did not provide diagnostic services were observed to have notable deficiencies in infrastructure. Conversely, facilities that conducted both cultures and antimicrobial susceptibility testing (AST) exhibited considerably higher scores. These results were found to be quite similar throughout the various areas that were evaluated. The primary obstacle to the successful application of susceptibility testing was the insufficiency of equipment. Allocation of resources toward equipment and infrastructure increases the capabilities for conducting antimicrobial susceptibility tests. This presents a significant opportunity for substantial progress in the field of antimicrobial resistance (AMR) diagnostics and surveillance within the country. The findings of this assessment will contribute to the advancement of sustainable laboratory-based AMR surveillance efforts in the country.

The Guest Editors would like to express their gratitude to all the authors and reviewers of this Research Topic, and acknowledge their hard work and dedication toward the area of biorisk management, laboratory acquired infections, and clinical containment. The Guest Editors believe that this work will encourage the generation of more knowledge and valuable research in the biosafety, biosecurity, and related fields.

## Author contributions

KA: Writing—original draft, Writing—review & editing. EM: Writing—review & editing. FK: Writing—review & editing.

